# Transcervical vs. Transcervical-Combined Surgical Approaches for Primary Parapharyngeal Space Tumors: A Systematic Review of Surgical and Functional Outcomes

**DOI:** 10.3390/cancers18040676

**Published:** 2026-02-19

**Authors:** Volodymyr Mavrych, Saniyah Shaikh, Hafsah Tajammul Khalifey, Safwaan Shaikh, Luqman Siddique, Thaabit Raziq, Anam Hashmi, Farah Abul Rub, Olena Bolgova

**Affiliations:** College of Medicine, Alfaisal University, Riyadh 11533, Saudi Arabia

**Keywords:** parapharyngeal space, transcervical approach, surgical outcomes, cranial nerve injury, systematic review, head and neck surgery, complications

## Abstract

Primary parapharyngeal space (PPS) tumors are rare neoplasms comprising 0.5% of head and neck tumors. Their complex anatomical location and proximity to critical neurovascular structures pose significant surgical challenges. While transcervical and transcervical-combined approaches represent the primary surgical techniques, no comprehensive systematic comparison exists. This systematic review compared surgical success, complications, functional outcomes, and recurrence rates between these approaches. Following PROSPERO registration (CRD420251037201), we searched PubMed, Cochrane, Web of Science, Google Scholar, and ScienceDirect without date restrictions. Independent dual screening identified retrospective cohort studies and case series comparing both approaches. Data extraction and risk of bias assessment employed standardized tools, with synthesis conducted per PRISMA 2020 guidelines using narrative analysis. Ten studies encompassing 505 patients with 508 tumors met inclusion criteria. Both approaches achieved excellent complete resection rates (95–100%). Transcervical approaches demonstrated lower overall complication rates (4.8–52.6%) versus transcervical-combined approaches (7.7–100%), though rates varied substantially by tumor type, and differences likely reflect case selection rather than approach-specific effects. Cranial nerve injuries (VII, X, XII) constituted predominant complications. Infratemporal fossa approaches showed the highest morbidity. Recurrence rates ranged from 0–30.3% without consistent patterns favoring either approach. Transcervical-combined approaches were essential for superior compartment extension and for skull base involvement. Both transcervical and transcervical-combined approaches achieve excellent tumor resection with acceptable morbidity when appropriately selected. For most benign PPS tumors, a transcervical approach may be preferred. Combined approaches may be considered in specific anatomic scenarios that require enhanced exposure, particularly when the superior compartment and skull base are involved, although the decision is always highly individual.

## Simple Summary

Tumors that grow in the parapharyngeal space, a small area deep in the neck near important blood vessels and nerves, are very rare but challenging to remove surgically. Surgeons can access these tumors through different routes, with the most common being a neck incision alone or a neck incision combined with additional cuts through other structures for better access. This study reviewed all available research comparing these two surgical approaches to determine which is safer and more effective. We analyzed 10 studies involving 505 patients to compare success rates, complications, and tumor recurrence between the approaches. Both methods successfully removed tumors in almost all cases. The simpler neck-only approach caused fewer complications overall, though complication rates varied depending on tumor characteristics. The main complications for both approaches involved injury to nerves controlling facial movement and swallowing. Transcervical-combined approaches were necessary when tumors extended to certain difficult-to-reach areas. Our findings help surgeons choose the best approach, with the decision being highly individual for each patient, generally recommending the simpler neck-only method for most benign tumors while reserving combined approaches for specific cases requiring greater surgical access.

## 1. Introduction

From the base of the skull to the larger cornu of the hyoid bone, the parapharyngeal space (PPS) is an anatomically complex potential space in the shape of an inverted pyramid [1]. Even for skilled surgeons, surgical access is extremely challenging due to the relatively small size and the presence of critical neurovascular structures within it [2,3]. PPS tumors are extremely uncommon; they make up about 0.5% of all head and neck neoplasms, of which about 80% are benign [4,5,6,7,8]. They constitute a diagnostic dilemma and a therapeutic challenge due to their rarity, pathogenic complexity, and frequently obscure clinical presentation. Salivary gland tumors and neurogenic tumors are the most prevalent histologies, with vascular and other diseases occurring occasionally [9,10].

The mainstay of treatment for PPS tumors continues to be surgical excision [9,11,12]. To navigate this intricate area, several surgical techniques have been developed over time, including transoral, transcervical, transparotid, transmandibular, and combinations of these techniques [13,14,15]. The transcervical technique has been emphasized as both adaptable and successful among the various surgical strategies developed for PPS tumors [16]. It allows superior vascular control and extensive exposure to the PPS, which is particularly helpful when cancers are located close to important vessels [17,18]. In surgical planning, preoperative imaging with CT or MRI is essential for defining tumor boundaries and their connections to critical structures [11,19]. In certain instances, further exposure through a transparotid extension or mandibulotomy might be necessary to guarantee safe dissection and total tumor removal. Although there are risks associated with each strategy, the final decision is influenced by the tumor’s size, location, and presumed pathology [7,20,21].

Although there are many different surgical options for PPS tumors, there is no universal agreement on the best course of action, particularly when transcervical access is not enough [21]. With differing findings regarding safety, recurrence rates, complication rates, and functional outcomes, the literature frequently focuses on institutional or individual choices [3,22]. Additionally, other studies indicate no discernible difference between the transcervical and combination techniques, with increased consequences such as Horner’s syndrome or cranial nerve palsies [8,13,23,24,25]. A thorough comparison of transcervical and transcervical-combined methods remains necessary, particularly with respect to postoperative complications, recurrence rates, and patient-centered outcomes, such as length of hospital stay and patient satisfaction.

The objective of this systematic review is to compare and critically assess the surgical and functional results of transcervical and transcervical-combined techniques for the excision of primary tumors in the parapharyngeal space. By evaluating available evidence, this review intends to:

- Evaluate and contrast the two methods’ recurrence and complication rates.
- Examine functional results, such as surgical healing, Horner’s syndrome, and nerve damage.
- Make evidence-based suggestions to direct surgical planning and enhance patient outcomes while managing PPS tumors.

## 2. Materials and Methods

### 2.1. Protocol Registration

This Systematic Review was prospectively registered in PROSPERO (CRD420251037201).

### 2.2. Inclusion Criteria

Studies were eligible if they included patients of any age with primary tumors of the parapharyngeal space, regardless of tumor histology. The intervention of interest was the transcervical approach, compared with a transcervical-combined approach defined as any technique extending beyond standard transcervical access, including transparotid extensions, transmastoid approaches, mandibulotomy, infratemporal fossa approaches, transoral robotic surgery, or endoscopic-assisted transcervical approaches. Eligible study designs included retrospective cohort studies, prospective cohort studies, case series with comparative arms, and clinical trials (randomized or non-randomized) comparing the two surgical approaches. Lastly, only published studies reported in the English language were included.

### 2.3. Exclusion Criteria

Studies were excluded if they included patients with metastatic or recurrent tumors in the parapharyngeal space, or patients with co-morbidities affecting the parapharyngeal region (e.g., neurofibromatosis, prior craniofacial surgery, congenital anomalies) that may influence surgical access, outcomes, or complication rates unless these subgroups were analyzed and reported separately. Studies focusing solely on non-surgical interventions (e.g., chemotherapy, radiotherapy) or those reporting only one surgical approach (e.g., only transcervical or only transcervical-combined approach) without a comparator group were also excluded. Additionally, studies that compared transcervical approaches to non-surgical treatments (e.g., radiotherapy, chemotherapy) or that failed to compare data on outcomes between the two approaches were excluded.

Single-patient case reports, editorials or commentaries, conference abstracts without full-text articles, reviews, animal or cadaveric studies, unpublished studies, the grey literature, or non-English language studies without an English abstract or translation were also excluded.

### 2.4. Search Strategy

A comprehensive literature search was conducted across PubMed, Cochrane, Web of Science, Google Scholar, and ScienceDirect, with no date restrictions. Search strategies were customized to match the indexing system and syntax requirements of each database. The following search formulas were applied:

PubMed: (“parapharyngeal space” [Title/Abstract] OR “parapharyngeal tumor*” [Title/Abstract] OR “parapharyngeal neoplasm*” [Title/Abstract]) AND (“transcervical” [Title/Abstract] OR “transcervical combined” [Title/Abstract] OR “mandibulotomy” [Title/Abstract] OR “skull base approach*” [Title/Abstract] OR “transoral robotic surgery” [Title/Abstract] OR “TORS” [Title/Abstract] OR “endoscopic” [Title/Abstract]) AND (“surgical outcome*” [Title/Abstract] OR “functional outcome*” [Title/Abstract] OR “complication*” [Title/Abstract] OR “nerve injury*” [Title/Abstract] OR “speech” [Title/Abstract] OR “swallowing” [Title/Abstract] OR “recurrence” [Title/Abstract] OR “quality of life” [Title/Abstract]).

Central Cochrane Library: (“parapharyngeal space” OR “parapharyngeal tumor*” OR “parapharyngeal neoplasm*”) AND (“transcervical” OR “mandibulotomy” OR “skull base” OR “transoral robotic surgery” OR “TORS” OR “endoscopic”).

Web of Science: TS = (“parapharyngeal space” OR “parapharyngeal tumor*” OR “parapharyngeal neoplasm*”) AND TS = (“transcervical” OR “mandibulotomy” OR “skull base” OR “transoral robotic surgery” OR “TORS” OR “endoscopic”) AND TS = (“surgical outcome*” OR “functional outcome*” OR “complication*” OR “nerve injury” OR “speech” OR “swallowing” OR “recurrence” OR “quality of life”).

Science Direct: “parapharyngeal space” AND (“transcervical” OR “mandibulotomy” OR “TORS” OR “endoscopic”) AND (“surgical outcomes” OR “complications” OR “nerve injury” OR “recurrence”).

Google Scholar: “parapharyngeal space tumor” transcervical “surgical outcomes” OR “functional outcomes” OR “nerve injury”.

### 2.5. Data Extraction

All search results were independently screened by two reviewers in a two-stage process (title/abstract and full-text screening). Any disagreements were resolved through discussion until consensus was achieved. The selection process adhered to PRISMA 2020 guidelines and is illustrated in a PRISMA flow diagram (Table S3).

Data extraction was performed independently by two reviewers using a standardized extraction form developed a priori based on the PICOS framework. The form captured study identifiers, characteristics, population characteristics, intervention and comparator details, outcome measures (both primary and secondary), quantitative results, qualitative feedback, and assessments of quality and bias. Discrepancies were resolved by consensus or the involvement of a third reviewer.

### 2.6. Risk of Bias Assessment

The risk of bias was assessed using the ROBINS-I tool for retrospective comparative/observational studies. At the same time, case series were evaluated using the NIH Quality Assessment Tool for Case Series Studies.

## 3. Results

### 3.1. Search Results

**Figure 1:**
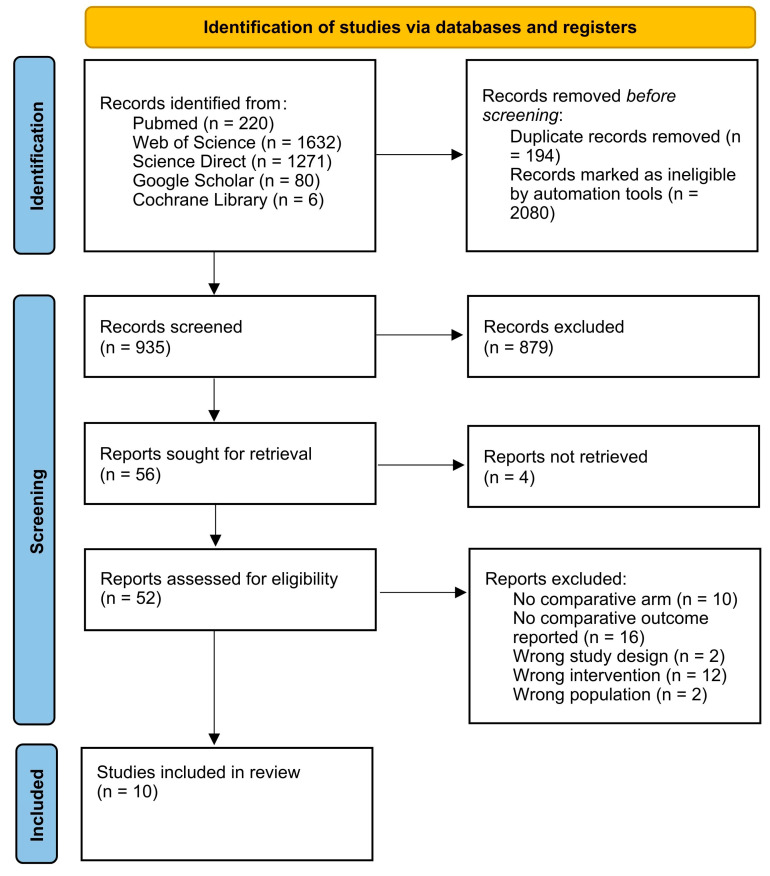
PRISMA 2020 flowchart depicting the study selection process.

A methodical search in PubMed, Web of Science, ScienceDirect, Google Scholar, and the Cochrane Library identified 3209 articles. After removing 194 duplicate articles and 2080 articles using the Rayyan web application, 935 articles were screened based on their titles and abstracts. Of these, 879 articles were excluded, resulting in 56 articles for full-text retrieval. Four articles could not be retrieved, leaving 52 articles for full-text screening. Following this, 42 articles were excluded, and ultimately, 10 articles were included in this study (Figure 1).

The 10 studies selected for inclusion in this systematic review were published between 2005 and 2020. Most of the studies were conducted in Italy [21,26,27,28,29], while the remaining were done in Mexico [30], Iran [1], US [31], Israel [32] and India [18]. All studies were retrospective studies consisting of patients with parapharyngeal space tumors. Sample size of patients ranged from 14 to 166 and included patients aged from 6 months to 80 years including males and females. Half of the included studies had patients with benign parapharyngeal space (PPS) tumors only [18,27,28,29,32], while the others included patients having either benign or malignant tumors [1,21,26,30,31]. Both transcervical and transcervical-combined approaches were employed across all included studies. The combined approaches incorporated parotidectomy, transparotid, transmastoid, transoral, transmandibular, laminectomy, and mandibulectomy techniques. Duration of follow-up ranged from 1 month to 16 years. Extracted information from these studies is shown in Tables S1 and S2.

### 3.2. Bias Assessment

**Figure 2:**
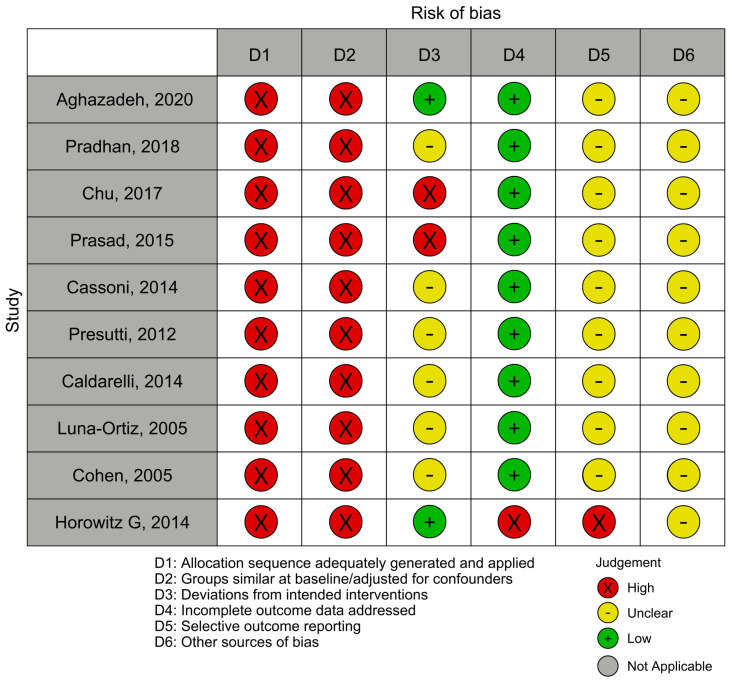
Risk-of-bias assessments were visualized using the robvis web application [1,18,21,26,27,28,29,30,31,32,33].

The overall body of evidence can be judged to carry a high risk of bias, although most domains across individual studies are rated as moderate; all included studies demonstrate at least two domains rated as high risk (Figure 2).

This higher risk is largely due to the retrospective design, potential confounding, and lack of standardized outcomes. Among these, Chu et al. is the most concerning, with a critical risk, whereas Caldarelli et al. represents the most balanced observational study, showing only moderate bias but with a smaller sample size [21,29]. In contrast, the case series consistently demonstrates moderate risk of bias, but they provide valuable data on surgical outcomes and complications. Smaller case series, such as those by Presutti, Pradhan, and Horowitz, add important context for specific techniques (e.g., avoiding mandibulotomy) [18,28,32]. However, the largest and most informative study is Cohen et al. (166 patients), which combines a large cohort with relatively lower bias and thus provides the strongest evidence for systematic reviews [31].

## 4. Discussion

This systematic review evaluated the surgical and functional outcomes of transcervical versus transcervical-combined approaches for primary parapharyngeal space (PPS) tumors across 10 studies, including 505 patients with 508 tumors. The evidence demonstrates that both surgical approaches achieve excellent rates of complete tumor resection, with transcervical approaches showing success rates of 95–100% and transcervical-combined approaches achieving 100% complete resection in most series. However, the approaches differ considerably in their complication profiles, with transcervical-combined techniques generally associated with higher rates of nerve injury and other morbidities.

The predominant tumor histologies identified across the included studies were pleomorphic adenomas (representing the majority of salivary gland tumors) and schwannomas (the most common neurogenic tumors), consistent with the established literature documenting that 70–80% of PPS tumors are benign. The prestyloid compartment was more commonly affected in most series, though the distribution varied by study and surgical center experience.

Complication rates demonstrated substantial variability across approaches and studies. Transcervical approaches alone showed complication rates ranging from 4.8% to 52.6%, with the higher rates primarily reported in series involving complex neurogenic tumors or vascular lesions requiring extensive dissection. In contrast, transcervical-combined approaches exhibited complication rates from 7.7% to 100%, with infratemporal fossa approaches uniformly showing the highest morbidity. Cranial nerve injuries, particularly affecting nerves VII, X, and XII, represented the most common complications across all surgical approaches. Recurrence rates were generally low across both approach types, ranging from 0% to 30.3%, with the highest recurrence observed in the Aghazadeh series using purely transcervical access for certain tumor types. Direct comparison was limited by variable follow-up duration and inconsistent surveillance protocols across studies.

### 4.1. Surgical Success and Complete Resection

The high rates of complete tumor resection (95–100%) achieved with both transcervical and transcervical-combined approaches underscore that surgical access method, when appropriately selected, effectively permits radical tumor removal. The transcervical approach demonstrated slight inferiority in one series (Chu: 95% vs. 100%), though this difference may reflect selection bias, with more challenging tumors allocated to transcervical-combined approaches [21]. Importantly, the achievement of complete resection appears less dependent on approach extensiveness than on meticulous surgical technique, tumor characteristics, and preoperative planning.

Several authors, including Presutti and Cassoni, advocate strongly for avoiding mandibulotomy even in large tumors (>5 cm), arguing that skilled surgeons can achieve adequate exposure through transcervical or transcervical-transparotid routes [27,28]. This philosophy prioritizes minimizing surgical morbidity while maintaining oncologic adequacy. The data support this approach for the majority of benign PPS tumors, though transcervical-combined approaches remain essential for specific anatomic scenarios, including superior compartment extension, skull base involvement, or significant vascular encasement.

### 4.2. Complications and Morbidity Profiles

The complication profiles revealed critical differences between approaches. Transcervical approaches demonstrated lower overall complication rates in most series, ranging from 4.8% (Cassoni, purely transcervical) to 33% (Luna-Ortiz) [27,30]. However, the nature and severity of complications varied considerably based on tumor type rather than approach alone. Neurogenic tumors, particularly vagal paragangliomas and schwannomas, were associated with higher cranial nerve injury rates regardless of surgical access method, as noted by Caldarelli and Luna-Ortiz [29,30]. This observation suggests that tumor biology and anatomic relationships to neural structures may be more deterministic of postoperative nerve function than the extent of surgical exposure. Histologic heterogeneity further complicates outcome interpretation. Paragangliomas, being vascular tumors, present distinct surgical challenges [31]. Pleomorphic adenomas vary in adherence and require meticulous capsular dissection [32]. Schwannomas often have clear dissection planes. These histology-specific factors influence complication rates independent of surgical approach, emphasizing the importance of anatomy-guided rather than histology-based approach selection.

Transcervical-combined approaches, particularly those incorporating mandibulotomy or infratemporal fossa extensions, invariably produced higher complication rates. Prasad reported 100% complication rates for the infratemporal fossa approach type A, consisting primarily of facial nerve deficits and conductive hearing loss [26]. Similarly, Chu documented cerebral stroke in 50% of mandibulotomy cases, though this catastrophic complication likely reflects vascular manipulation rather than mandibular osteotomy per se [21]. These findings emphasize that combined approaches may be reserved for tumors where transcervical access would be inadequate, accepting the increased morbidity as necessary to achieve safe and complete resection. However, studies reported grouped outcomes precluding detailed subgroup analysis by transcervical-combined approach type.

Horner’s syndrome emerged as a frequent complication, particularly after removal of sympathetic chain schwannomas or extensive dissection in the poststyloid compartment. Aghazadeh reported Horner’s syndrome in 12.1% of transcervical cases and 40% of transcervical-combined approach cases [1]. While generally well-tolerated by patients, Horner’s syndrome represents permanent autonomic dysfunction and should be considered as a factor in surgical decision making and patient counseling.

First bite syndrome, though infrequently reported, appeared in several series utilizing transparotid extensions [32]. This painful complication results from loss of sympathetic innervation to the parotid gland and typically resolves within months, but it may significantly affect quality of life during the recovery period.

Non-neurological complications received limited attention in the included studies. Vascular complications, including postoperative hemorrhage, were reported but inconsistently documented [13,24]. Cosmetic outcomes were documented in only three studies [27,28,29], with most focusing solely on functional rather than aesthetic results. This represents a critical gap in the literature, as patients often prioritize cosmetic and quality-of-life outcomes.

### 4.3. Cranial Nerve Injuries

Cranial nerve injuries represented the most clinically significant complications across all surgical approaches. The facial nerve (CN VII), hypoglossal nerve (CN XII), and vagus nerve (CN X) were most frequently affected. The pattern of nerve injury correlated strongly with tumor location and histology:

Facial nerve injuries occurred primarily with prestyloid tumors requiring parotidectomy or transparotid approaches. Presutti and Caldarelli reported mandibular branch weakness in multiple cases, though most demonstrated complete recovery within 3–6 months [28,29]. Prasad documented higher rates of facial nerve dysfunction with infratemporal fossa approaches, reflecting the more extensive facial nerve manipulation required for these complex exposures [26].

Vagus nerve dysfunction, manifesting as vocal cord paralysis and dysphagia, occurred predominantly during resection of poststyloid neurogenic tumors. Caldarelli noted that vagal paragangliomas frequently required sacrifice of the nerve due to tumor encasement [29]. When vagal preservation was achieved, patients typically experienced only hoarseness without aspiration, whereas vagal sacrifice led to more severe swallowing difficulties that required prolonged rehabilitation.

Hypoglossal nerve injuries appeared across multiple series, with Presutti reporting CN XII deficits in four patients [28]. Notably, vocal cord paralysis demonstrated better recovery potential than hypoglossal nerve injuries, with the latter often resulting in permanent tongue deviation and articulation difficulties.

### 4.4. Recurrence Patterns

Recurrence rates varied dramatically across studies, ranging from 0% in several series to 30.3% in Aghazadeh’s transcervical group [1]. This variation likely reflects differences in tumor histology, completeness of capsular excision, and adequacy of follow-up. Pleomorphic adenomas, even when benign, carry inherent recurrence risk if capsular disruption occurs during dissection, as noted by Chu [21]. The Aghazadeh series, which demonstrated the highest recurrence rate, employed transcervical approaches for all recurrent cases, suggesting that inadequate initial exposure may have compromised complete tumor removal in these patients [1].

Several authors emphasized that recurrence is more closely associated with surgical technique (specifically, avoiding capsular rupture and achieving complete excision with adequate margins) than with the choice of approach. However, transcervical-combined approaches may facilitate more controlled dissection in challenging anatomic scenarios, potentially reducing inadvertent capsular violation.

### 4.5. Approach Selection Based on Tumor Characteristics

The accumulated literature suggests potential patterns in surgical approach selection, though these derive from retrospective case series rather than prospective comparative studies. Prasad et al. proposed a compartment-based algorithmic framework dividing the parapharyngeal space into lower, middle, and upper compartments [26].

Based on this framework and supporting observations from multiple series, the following patterns have been described:

- Transcervical approach has been utilized for lower compartment tumors and poststyloid neurogenic tumors without superior extension [26,31].
- Transcervical-transparotid approach has been employed for deep lobe parotid tumors, middle compartment prestyloid masses [21,24,26,27,31,32].
- Transcervical-transmastoid approach has been described for upper compartment tumors with posterior extension and selected vascular tumors involving the jugular bulb [26,31].
- Mandibulotomy and infratemporal fossa approaches have generally been reserved for malignant tumors and recurrent cases [24,27,28,31].

Importantly, multiple authors demonstrate that tumor size alone does not dictate approach selection, with successful transcervical removal of large tumors reported [24,27,28]. The decision depends more critically on vertical extent, skull base involvement, and relationship to neurovascular structures [24,26,31]. This framework represents a conceptual guide emphasizing the least morbid approach adequate for complete resection [27,28,32], though tumors frequently cross compartments and individual factors must inform final decision making [26]. The strength of evidence remains limited by retrospective design, lack of standardized outcomes, and selection bias. 

### 4.6. Comparison with the Existing Literature

The findings of this systematic review align with and extend the previous literature on PPS tumor management. Earlier systematic reviews by Kuet (2015) and Riffat (2014) similarly emphasized the heterogeneity of PPS tumors and variability in surgical approaches [7,8]. However, those reviews did not specifically compare transcervical versus transcervical-combined approaches in a structured manner.

López et al. (2019) provided contemporary management recommendations for PPS tumors but without systematic evidence synthesis comparing approach-specific outcomes [11]. Our review builds upon this work by quantifying complication and recurrence rates for specific surgical techniques across multiple centers.

The debate regarding mandibulotomy necessity, prominently featured in the Presutti series, reflects evolving surgical philosophy [28]. Historically, many surgeons advocated routine mandibulotomy for adequate PPS exposure [34]. However, accumulating evidence from experienced centers demonstrates that skilled surgeons can avoid mandibulotomy in the vast majority of benign cases, reserving this more morbid approach for malignant or particularly challenging tumors.

Emerging minimally invasive techniques, including transoral robotic surgery (TORS) and endoscopic-assisted transcervical approaches, show promise for selected PPS tumors. Chu’s series included seven TORS cases without major complications, suggesting this approach may eventually occupy a niche for small, well-circumscribed prestyloid tumors [21]. However, the limited data and highly selected patient populations currently preclude definitive conclusions regarding the utility of TORS in PPS surgery. Contemporary surgical practice increasingly emphasizes minimally invasive options where anatomically feasible [16,18]. However, transcervical approaches remain the workhorse for most parapharyngeal space tumors, with transcervical-combined approaches reserved for specific anatomic challenges.

### 4.7. Clinical Implications and Recommendations for Practice

Given the methodological limitations of the available evidence, the following represents a suggested framework that synthesizes the available literature and expert opinions rather than providing evidence-based guidelines. No high-quality evidence currently exists regarding optimal surveillance protocols; accordingly, these recommendations reflect common practice patterns rather than definitive guidance. Approach selection requires individualized assessment based on tumor anatomic characteristics. Preoperative imaging with CT or MRI provides initial guidance for surgical planning [26,28]. However, intraoperative findings may necessitate modification of the approach, and the surgeon’s experience influences the selection of technique. This limits the generalizability of algorithmic approaches to all surgical settings. Transcervical approach may be considered for lower compartment tumors and prestyloid lesions without superior extension. Transcervical-combined approaches may be considered for superior compartment extension, skull base involvement, or when transcervical access proves inadequate. The decision should incorporate tumor size, location, histology, vascular relationships, and surgeon experience. Preoperative patient counseling must address approach-specific risks, particularly cranial nerve injury potential. For poststyloid neurogenic tumors, the possibility of vagal sacrifice and resulting voice/swallowing dysfunction requires detailed discussion.

Surgical centers managing PPS tumors should maintain multidisciplinary teams including head and neck surgeons, skull base surgeons, and neurosurgeons to optimize approach selection for complex cases.

Long-term surveillance is essential, particularly for pleomorphic adenomas, where late recurrence may occur. Regular clinical examination and interval imaging (MRI at 6, 12, 24 months, then biannually) should be considered as standard practice.

### 4.8. Limitations and Methodological Considerations

This systematic review possesses several notable strengths. First, it employed rigorous methodology with prospective protocol registration (PROSPERO), comprehensive multi-database searching without date restrictions, and independent dual review for study selection and data extraction. Second, this review focused on comparing transcervical and transcervical-combined approaches, thereby filling a defined gap in the literature. Third, quality assessment using validated tools (e.g., ROBINS-I, NIH Quality Assessment Tool) provides transparency into evidence reliability. Narrative synthesis was appropriate given the heterogeneity in study designs, populations, and outcome definitions precluding meta-analysis, consistent with Cochrane Handbook guidance for systematic reviews. Fourth, the inclusion of ten studies with 505 patients provides a reasonable sample size, though heterogeneity limits meta-analytic synthesis.

Several limitations constrain the conclusions. The most critical limitation of this systematic review is confounding by indication. Trans-cervical combined approaches are typically selected for tumors with greater anatomic complexity, including superior compartment extension, skull base involvement, or significant vascular encasement [26,31]. Therefore, observed differences in complication rates primarily reflect case selection rather than approach-specific effects. Second, substantial heterogeneity exists across studies regarding tumor histologies, sizes, and anatomic locations. Some series included only benign tumors, while others incorporated malignancies. Tumor size varied from 2 cm to >10 cm across studies. This heterogeneity prevented meta-analytic pooling and limits generalizability of specific complication rate estimates.

Third, outcome definitions varied substantially across studies. Complications were inconsistently categorized as transient versus permanent, and no validated quality-of-life instruments were employed. This heterogeneity limits the interpretation of reported complication ranges. Follow-up durations ranged from 1 month to 16 years, with inadequate long-term data in several series to accurately assess late recurrence. Fourth, publication bias likely affects the literature, with centers achieving excellent outcomes more motivated to report results. The identified studies primarily originated from high-volume specialized centers, potentially overestimating generalized surgical outcomes.

Fifth, this review did not assess emerging techniques including TORS and endoscopic approaches, in detail, as insufficient comparative data currently exist. Future updates should incorporate these evolving modalities as evidence accumulates.

Finally, the uniformly high risk of bias substantially limits confidence in the synthesized findings. All included studies showed serious or critical concerns in multiple ROBINS-I domains, primarily due to confounding, participant selection, and outcome measurement issues. This represents low-certainty evidence suitable for hypothesis generation but not definitive practice guidance.

### 4.9. Directions for Future Research

Several research priorities emerge from this systematic review:

Prospective comparative studies, ideally multi-center registries using standardized outcome definitions, would substantially improve evidence quality. While randomization may be impractical given tumor rarity and ethical considerations regarding approach selection, well-designed prospective cohorts with propensity score matching could minimize confounding.

Standardized outcome reporting using validated instruments for cranial nerve function, voice, swallowing, and quality of life would facilitate meaningful comparison across studies.

Long-term follow-up data, particularly beyond 5 years, is needed to accurately characterize late recurrence risks and functional recovery trajectories. Pleomorphic adenomas demonstrate recurrence potential decades after resection, necessitating extended surveillance.

Cost-effectiveness analyses comparing approaches would inform value-based surgical decision making, considering not only direct procedural costs but also downstream expenses related to complications, rehabilitation, and hospital stay duration.

Investigation of novel techniques including TORS, endoscopic-assisted transcervical approaches, and intraoperative navigation/image guidance should be prioritized. These modalities may expand the scope of minimally invasive surgery for PPS tumors.

Molecular and genetic profiling to predict tumor behavior and recurrence risk could enhance personalized surgical planning. For example, identifying pleomorphic adenomas at high risk for malignant transformation might justify more extensive initial resection.

Surgical technique standardization and training research could identify optimal methods for nerve identification and preservation. Simulation-based training for PPS surgery might reduce complication rates, particularly at lower-volume centers.

## 5. Conclusions

This systematic review provides comprehensive evidence comparing transcervical and transcervical-combined surgical approaches for primary parapharyngeal space tumors. Transcervical and transcervical-combined approaches both achieve high complete resection rates for parapharyngeal space tumors. Complication rates were associated with lower reported morbidity for transcervical approaches, though differences likely reflect case selection rather than approach-specific effects. Transcervical-combined approaches remain essential for specific anatomic scenarios but should not be routinely employed for uncomplicated benign PPS tumors. Surgical approach selection should be guided primarily by tumor anatomic characteristics rather than assumptions about approach-specific morbidity. The uniformly high risk of bias across all included studies limits definitive conclusions and highlights the need for higher-quality comparative research. Cranial nerve injury represents the predominant complication risk across all approaches, necessitating careful preoperative planning and meticulous surgical technique. Future research should focus on prospective comparative studies with standardized outcome reporting to further refine surgical decision-making algorithms and optimize patient outcomes.

## Data Availability

No new data were created or analyzed in this study.

## Supplementary Materials

The following supporting information can be downloaded at: https://www.mdpi.com/article/10.3390/cancers18040676/s1, Table S1: Main outcomes reported; Table S2: Additional outcomes reported; Table S3: PRISMA 2020 Checklist.

## Abbreviations

The following abbreviations are used in this manuscript:

PSS	Parapharyngeal Space
CN	Cranial Nerve (followed by Roman numerals: VII, X, XII)
CT	Computed Tomography
MRI	Magnetic Resonance Imaging
TORS	Transoral Robotic Surgery
PROSPERO	International Prospective Register of Systematic Reviews
PRISMA	Preferred Reporting Items for Systematic Reviews and Meta-Analyses
PICOS	Population, Intervention, Comparator, Outcome, Study design
ROBINS-I	Risk Of Bias In Non-randomized Studies of Interventions
NIH	National Institutes of Health
