# Supplementary material for: Transcervical vs. Transcervical-Combined Surgical Approaches for Primary Parapharyngeal Space Tumors: A Systematic Review of Surgical and Functional Outcomes
Source: Cancers (Basel). 2026 Feb 19;18(4):676. doi: 10.3390/cancers18040676 (PMC12939373; doi:10.3390/cancers18040676)
Supplement: Supplementary file 1 [file cancers-18-00676-s001.zip › Table S1.pdf]

Table S1. Main outcomes reported

Study Reference	Type of Tumor	Sample size	Approach: Transcervical Only			Type of transcervical - combined approach used	Approach: Transcervical - Combined		
			Surgical success	Complications	Recurr ence		Surgical success	Complications	Recurrence
Chu, 2017, [21]	Parapharyngeal space (PPS) tumors	53	21/22 (95%)	2/22 (9%: 1 facial nerve injury, 1 capsule rupture)	4/22 (18%)	TCP (Transcervical + Parotidectomy)	17/17 (100%)	4/17 (24%) - 1 partial facial nerve impairment, 3 facial nerve resections	0/17 (0%)
						TCM (Transcervical + Mandibulotomy)	2/2 (100%)	1/2 (50%) - Cerebral stroke	2/2 (100%)
						TORS + TC (Transoral Robotic + Transcervical)	1/1 (100%)	1/1 (100%) - capsular disruption	1/1 (100%)
Prasad, 2015, [26]	46 patients, 48 tumors	Prestyloid (12, 25%), Poststyloid (36, 75%)	19/19 (100%)	10/19 (52.6%) - Facial nerve (inferior branch) transient dysfunction, Hypoglossal nerve palsy, Vagus nerve palsy, CSF leak	0/19 (0%)	Transcervical–Transparotid	13/13 (100%)	1/13 (7.7%) - Facial nerve (marginal branch) transient paresis	0/13 (0%)
						Transcervical–Transmastoid	6/6 (100%)	2/6 (33.3%) - Lower cranial nerve deficits	0/6 (0%)
						Infratemporal fossa approach-type A	6/6 (100%)	6/6 (100%) - Facial nerve deficits (HB II–III), conductive hearing loss	0/6 (0%)
						Infratemporal fossa approach-type B	1/1 (100%)	1/1 (100%) - Lower cranial nerve deficits	0/1 (0%)
						Infratemporal fossa approach-type D	2/2 (100%)	2/2 (100%) - Facial nerve deficits (HB VI → III), CSF leak (1 patient), lower cranial nerve deficits	1/2 (50%) - pleomorphic adenoma → carcinoma ex-

									pleomorphic adenoma
Cassoni, 2014, [27]	60	Benign PPS	21/21 (100%)	1/21 (4.8%) - Marginal mandibular nerve palsy	0/21 (0%)	Transcervical + Mandibulotomy	3/3 (100%)	1/3 (33.3%) - V3 nerve sacrifice	0/3 (0%)
						Combined approaches (TCA + Transoral / Others)	6/6 (100%)	1/6 (16.7%) - Marginal mandibular + V3 damage	1/6 (16.7%)
Presutti, 2012, [28]	18	Benign PPS tumors	7/7 (100%)	7/7 (100%) - CN XII deficit (4), vocal cord paralysis (3), mandibular branch VII palsy (1)	1/7 (14%)	Transcervical–transparotid (TCTP)	11/11 (100%)	4/11 (36.4%) - CN XII deficit (1), vocal cord paralysis (1), mild dysphagia (2)	1/11 (9%)
Luna-Ortiz, 2005, [30]	Benign 16 (76%), Malignant 5 (24%)	21	12/12 (100%)	4/12 (33%) - nerve injuries (X, XII)	0/12 (0%)	Transcervical + Parotidectomy (4)	4/4 (100%)	0/4 (0%)	0/4 (0%)
						Transcervical + Transoral (2)	2/2 (100%)	1/2 (50%) - vagus nerve injury	0/2 (0%)
						Transcervical + Segmental Mandibulectomy	1/1 (100%)	0/1 (0%)	1/1 (100%) - recurrence at 11 years
						Transcervical + Laminectomy	0/1 (R1, residual tumor)	1/1 (100%) - C2 sensory deficit	0/1 (0%)
Caldarelli, 2014, [29]	Benign (100%)	21	14/14 (100%)	4/14 (28.5%) - 3 unilateral vocal cord paralysis, 1 Horner's syndrome	0/14 (0%)	Transcervical + Transparotid (3)	3/3 (100%)	1/3 (33.3%) - temporary mandibular branch VII dysfunction (resolved)	0/3 (0%)
						Transcervical + Transparotid + Transmandibular	1/1 (100%)	1/1 (100%) - temporary mandibular branch VII dysfunction	0/1 (0%)

Aghazadeh, 2020, [1]	78 tumors.<br>Pleomorphic adenoma: 61 (78.2%)<br>Schwannoma: 11 (14.1%)<br>Lipoma: 4 (5.1%)<br>Neurofibroma: 2 (2.6%)<br><br>Pre-styloid: 65 (83.3%)<br>Post-styloid: 13 (16.7%)	78	33/33 (100%)	4/33 (12.1%) Horner's syndrome<br>2/33 (6.1%) hematoma<br>3/33 (9%) CN X palsy	10/33 (30.3%)	Not specified	10/10 (100%)	4/10 (40%) Horner's syndrome<br>1/10 (10%) Hematoma	None
Cohen, 2005, [31]	166 PPS masses.<br><br>21 (12.7%) malignant<br><br>145 (87.3%) benign<br><br>76 (45.8%) vascular<br><br>69 (41.6 %) involving the skull base	166				TCTP	20/20 (100%)	6/20 (30%) -CN VII paresis	
Pradhan, 2018, [18]	14 patients	14	14/14 (100%)	3/10 (30%)	None reported	TCTOA - 2 (14.3%)	4/4 (100%)	TCTOA - Marginal mandibular palsy + wound infection	None reported
	28.57% Pre-styloid			2 cases: marginal mandibular palsy		TCTMA - 1 (7.14%)		TCTMA - Grade III facial palsy	
	71.42% Post-styloid			1 case: CN X and ICA damage		TCTPA - 1 (7.14%)			
Horowitz, 2014, [32]	29 patients	29	15/15 (100%)	1/15 (6.7%)	0/15 (0%)	TCTPA - 13 (44.8%)	14/14 (100%)	TCTPA -<br>CN VII paralysis, infection and first bite syndrome<br>TCTMA - None	None reported
	29 (100%) Pleomorphic adenoma					TCTMA - 1 (3.45%)			
