# Supplementary material for: Transcervical vs. Transcervical-Combined Surgical Approaches for Primary Parapharyngeal Space Tumors: A Systematic Review of Surgical and Functional Outcomes
Source: Cancers (Basel). 2026 Feb 19;18(4):676. doi: 10.3390/cancers18040676 (PMC12939373; doi:10.3390/cancers18040676)
Supplement: Supplementary file 1 [file cancers-18-00676-s001.zip › Table S2.pdf]

Table S2. Additional outcomes reported.

Study Reference	Approach: Transcervical Only					Approach: Transcervical - Combined				
	Operative time	Postoperative length of stay	Cosmetic impact	Quality of life	Blood loss	Operative time	Postoperative length of stay	Cosmetic impact	Quality of life	Blood loss
Chu, 2017, [21]	Not specifically reported	Variable - Generally shorter than other approaches	Good cosmetic outcome	Preserved	Occasional bleeding/capsule rupture	Not reported	Longer with TCM	Impairment with parotidectomy	Reduced in TCM	Occasional bleeding/capsule rupture
Prasad, 2015, [26]	Not specifically reported	Not specifically reported	Minimal cosmetic morbidity reported	Better functional outcomes compared to anterior approaches	Bleeding noted	Not specifically reported	Not specifically reported	Cosmetic morbidity low - facial nerve deficits in some combined approaches	Not numerically assessed but functional recovery reported	Significant blood loss
Cassoni, 2014, [27]	Not specifically reported	Not specifically reported	Transcervical incision 2–3 cm below mandible, minimal visible scarring	Not numerically assessed, but safe removal even for large PPS tumors	Risk of hemorrhage noted	Not specifically reported	Not specifically reported	Cosmetic morbidity remained low	1 V3 sacrifice, 1 case of V3 + marginalis damage	Risk of hemorrhage noted
Presutti, 2012, [28]	Not specifically reported	5–11 days	Minimal scar	1 permanent vocal cord paralysis	Not reported	Not specifically reported	3–17 days	1 temporary mandibular branch paresis	3 permanent vocal cord paralysis	Not reported
Luna-Ortiz, 2005, [30]	Not specifically reported	Not specifically reported	Good cosmetic outcomes	3 vagus nerve palsies	Not reported	Not specifically reported	Not specifically reported	Good cosmetic outcomes	1 vagus nerve palsy, 1 hypoglossal paralysis	Not reported
Caldarelli, 2014, [29]	Not specifically reported	Not specifically reported	Good cosmetic outcomes	3 vocal cord paralyses, 1 Horner's syndrome, 6	Controlled	Not specifically reported	Not specifically reported	Good cosmetic outcomes	2 temporary mandibular branch VII dysfunctions	Controlled

				transient hoarseness/dysphagia						
Aghazadeh, 2020, [1]	Not specifically reported	Mean 3.69	Small scar	Not specifically reported	Not specifically reported	Not specifically reported	Mean 4.9	Small scar	Not specifically reported	Not specifically reported
Cohen, 2005, [31]	Not specifically reported	Not specifically reported	Good cosmetic outcomes	Facial nerve weakness 7.8%	Not specifically reported	Not specifically reported	Not specifically reported	Good cosmetic outcomes	Vocal cord paralysis 29.3%, palatal weakness 19.7%,	Not specifically reported
Pradhan, 2018, [18]	Not specifically reported	Not specifically reported	Facial nerve injury in 3 patients	Not specifically reported	Internal carotid injury in 1 patient	Not specifically reported	Not specifically reported	Marginal mandibular/facial nerve injury in 2 patients, wound infection in 1 patient	Not specifically reported	Not specifically reported
Horowitz, 2014, [32]	Not specifically reported	Not specifically reported	Good cosmetic outcomes	Not specifically reported	Hemorrhage in 1 patient	Not specifically reported	Not specifically reported	Marginal mandibular/facial nerve injury (1 Transparotid), wound infection (1 Transparotid), first bite syndrome (1 Transparotid)	Not specifically reported	Not specifically reported
