# Supplementary material for: Transcervical vs. Transcervical-Combined Surgical Approaches for Primary Parapharyngeal Space Tumors: A Systematic Review of Surgical and Functional Outcomes
Source: Cancers (Basel). 2026 Feb 19;18(4):676. doi: 10.3390/cancers18040676 (PMC12939373; doi:10.3390/cancers18040676)
Supplement: Supplementary file 1 [file cancers-18-00676-s001.zip › Table S3 PRISMA 2020 Checklist.pdf]

PRISMA 2020 Checklist

Section and Topic	Item #	Checklist item	Location where item is reported
**TITLE**			
Title	1	"Transcervical vs Transcervical-Combined Surgical Approaches for Primary Parapharyngeal Space Tumors: A Systematic Review of Surgical and Functional Outcomes" - clearly identifies this as a systematic review	Page 1
**ABSTRACT**			
Abstract	2	Abstract with background, methods, results, and conclusions	Page 1
**INTRODUCTION**			
Rationale	3	Introduction - Four paragraphs describing PPS tumor rarity (0.5%), anatomical complexity, surgical challenges, lack of consensus on approaches, and conflicting literature on transcervical vs combined approaches	Page 2
Objectives	4	Introduction - Three bullet points: evaluate recurrence/complication rates, examine functional outcomes, make evidence-based suggestions	Page 2
**METHODS**			
Eligibility criteria	5	Section 2.2: primary PPS tumors, any age, transcervical vs transcervical-combined, comparative outcomes, retrospective/prospective cohorts, English language. Section 2.3: exclusion of metastatic/recurrent tumors, co-morbidities, single approach studies, case reports, conference abstracts, reviews, animal studies, unpublished studies, grey literature	Pages 3
Information sources	6	Section 2.4, first sentence - "PubMed, Cochrane, Web of Science, Google Scholar, and ScienceDirect databases, without restrictions on the search date"	Page 3
Search strategy	7	Complete search formulas for PubMed, Cochrane, Web of Science, ScienceDirect, and Google Scholar with all terms and Boolean operators	Pages 3-4
Selection process	8	Section 2.5, first paragraph - "two reviewers in a two-stage process (title/abstract and full-text screening)," "independently screened," "disagreements were resolved through discussion," "PRISMA 2020 guidelines," flow diagram referenced	Page 4
Data collection process	9	Section 2.5, second paragraph - "three reviewers using a standardized extraction form," "independently," "PICOS framework," "discrepancies resolved by consensus or the involvement of a third reviewer"	Page 4
Data items	10a	Objectives section mention of outcomes (complication rates, functional results, recurrence rates)	Page 2
	10b	Section 2.5, second paragraph -"study identifiers, characteristics, population characteristics, intervention and comparator details, outcome measures (both primary and secondary), quantitative results, qualitative feedback, and assessments of quality and bias"	Page 4
Study risk of bias assessment	11	Section 2.6 - "ROBINS-I tool for retrospective comparative/observational studies" and "NIH Quality Assessment Tool for Case Series Studies"	Page 4-5
Effect measures	12	Discussion, first paragraph - "complete resection rates (95-100%)," "complication rates ranging from 4.8% to 52.6%," "recurrence rates ranged from 0% to 30.3%" - proportions and percentages used	Page 7
Synthesis methods	13a	Section 2.5 and Page 5, Section 3.1 - Studies comparing transcervical vs transcervical-combined approaches	Page 4
	13b	Section 2.5 - Standardized extraction form based on PICOS	Page 4
	13c	Section 3.1 - References to "Fig. 1" (PRISMA flowchart), and Page 6 - "Fig. 2" (Risk of bias), Tables 1 and 2 mentioned	Pages 5-6
	13d	Section 2.5 - "PRISMA 2020 guidelines" for selection; Page 11, Section 4.9 - "heterogeneity prevented meta-analytic pooling"	Pages 4, 13
	13e	Section 4.9, third paragraph - "tumor histologies, sizes, and anatomic locations," "outcome definitions and reporting varied considerably"	Pages 11
	13f	Section 4.9 - no formal sensitivity analyses conducted	Page 11
Reporting bias assessment	14	Section 4.9, fifth paragraph - "publication bias likely affects literature"	Page 11
Certainty	15	Section 4.9 - GRADE approach was used to assess certainty of evidence	

Section and Topic	Item #	Checklist item	Location where item is reported
assessment			
**RESULTS**			
Study selection	16a	Results of the search and selection process, from the number of records identified in the search to the number of studies included in the review, is shown on the flow diagram.	Page 5
	16b	Risk-of-bias assessments details were visualized on Figure 2	Page 6
Study characteristics	17	Section 3.1, second paragraph - "10 studies published between 2005 and 2020," countries, study designs, sample sizes (14-166), ages (6 months-79 years), tumor types, approaches, follow-up (1 month-16 years), with references to Tables 1 and 2	Page 5
Risk of bias in studies	18	Section 3.2 - "moderate risk of bias," "observational comparative studies ranged from serious to critical risk (Fig.2)," specific studies discussed (Chu et al. critical risk, Caldarelli et al. moderate, Cohen et al. 166 patients)	Page 6
Results of individual studies	19	Discussion section - Study-specific results discussed throughout with references to Tables 1-2	Pages 7-10
Results of syntheses	20a	Section 3.2 - All retrospective, moderate to serious risk; Section 4.9, second paragraph - "All included studies were retrospective case series or observational cohorts, introducing inherent selection bias"	Page 6, 11
	20b	Section 4, first paragraph - "complete resection rates (95-100%)" both approaches; third paragraph - "Transcervical approaches alone showed complication rates ranging from 4.8% to 52.6%" vs "transcervical-combined approaches exhibited complication rates from 7.7% to 100%"; "Recurrence rates...0% to 30.3%"	Page 7
	20c	Section 4.9, third paragraph - "tumor histologies, sizes, and anatomic locations," size variation "2 cm to >10 cm," some series benign only vs mixed, outcome definition variations, follow-up 1 month to 16 years	Page 11
	20d	Section 4.9 - no formal sensitivity analyses conducted	
Reporting biases	21	Section 4.9, fifth paragraph - "publication bias likely affects literature, with centers achieving excellent outcomes more motivated to report results. The identified studies primarily originated from high-volume specialized centers, potentially overestimating generalized surgical outcomes"	Page 11
Certainty of evidence	22	Risk-of-bias assessments details were visualized on Figure 2	
**DISCUSSION**			
Discussion	23a	Section 4.7 - "Comparison with Existing Literature" discussing Kuet (2015), Riffat (2014), López et al. (2019), mandibulotomy debate, emerging TORS techniques	Pages 7-10
	23b	Section 4.9, paragraphs 2-6 - Retrospective designs with selection bias, heterogeneity in tumor characteristics, inconsistent outcome definitions, variable follow-up (1 month-16 years), publication bias from specialized centers, moderate to serious risk of bias	Page 11
	23c	Section 4.9, paragraph 3 - "heterogeneity prevented meta-analytic pooling"; paragraph 4 - "outcome definitions and reporting varied considerably," "inadequate long-term data," "functional outcome measures were inconsistently reported"; paragraph 6 - "the review did not assess emerging techniques including TORS and endoscopic approaches in detail"	Page 11
	23d	Section 4.8 - Clinical practice recommendations (6 points: individualized approach, transcervical as default, mandibulotomy reservations, preoperative counseling, multidisciplinary teams, long-term surveillance); Section 4.10 - "Directions for Future Research"	Pages 10-12
**OTHER INFORMATION**			
Registration and protocol	24a	Section 2.1 - "This Systematic Review was prospectively registered in PROSPERO (CRD420251037201)"	Page 3
	24b	Section 2.1 - "This Systematic Review was prospectively registered in PROSPERO (CRD420251037201)"	Page 3
	24c	There are no any amendments to information provided at registration or in the protocol	Page 3

PRISMA 2020 Checklist

Section and Topic	Item #	Checklist item	Location where item is reported
Support	25	This research received no funding	Page 12
Competing interests	26	The authors declare that the research was conducted in the absence of any commercial or financial relationships that could be construed as a potential conflict of interest.	Page 13
Availability of data, code and other materials	27	No new data were created or analyzed in this study	Page 12

From: Page MJ, McKenzie JE, Bossuyt PM, Boutron I, Hoffmann TC, Mulrow CD, et al. The PRISMA 2020 statement: an updated guideline for reporting systematic reviews. BMJ 2021;372:n71. doi: 10.1136/bmj.n71. This work is licensed under CC BY 4.0. To view a copy of this license, visit https://creativecommons.org/licenses/by/4.0/
